# Matrix or Ring Fillings

**Published:** 1890-11

**Authors:** L. C. Bryan

**Affiliations:** Basel, Switzerland


### International Dental Journal.

Vol. XI. November, 1890. No. 11.

### Original Communications.[1]

### MATRIX OR RING FILLINGS.[2]

BY DR. L. C. BRYAN, BASEL, SWITZERLAND.

One of the most important contributions to practical dental art literature is the article read by Dr. Dwight M. Clapp before the New York Odontological Society, and published in the *Dental Cosmos* of December, 1888, on combination fillings of gold and amalgam finished at one sitting. The clear and exhaustive manner in which Dr. Clapp presents his arguments in favor of combining gold and amalgam in large compound cavities in the molars cannot fail to convince thoughtful members of the profession that there is much of value in his suggestions. A subject of such universal importance, covering cases that are daily presented in the practice of every dentist, should receive something more than a passing notice. The subject should be more thoroughly discussed and greater attention be called to the importance of the method.

The matrix suggested by the doctor is novel and ingenious, and adds another to the long list of recent inventions in this department. However, all of these half matrices require more or less

[1] The editor and publishers are not responsible for the views of authors of papers published in this department, nor for any claim to novelty, or otherwise, that may be made by them. No papers will be received for this department that have appeared in any other journal published in the country.

[2] Read at the monthly meeting of the New York Odontological Society, June 17, 1890.

skill in their adjustment, and, considering the large variety of cases requiring the use of matrices, none have proved universally applicable. The ideal matrix should be as simple of application as a ring to one's finger, and all those which require screws, wrenches, threads, and clamps are unsatisfactory in that the general practitioner finds the skill and time required, and the difficulty of adjustment greater than the benefit resulting from their use. The ideal matrix then should consist of a simple ring, so thin as to pass readily between the most crowded teeth without disturbing their position but slightly, and without bending upon itself in its adjustment. It should be exceedingly flexible and springy, so that it may be bent into any position and return to its original form on the removal of pressure. It should not stretch or belly out when force is applied to it. It should be ready-made and at hand; of proper width and height, without necessity of adjusting; it should be possible to place it on a broken-down tooth of any degree of dilapidation, with or without remaining enamel walls. It should be easily removed after the completion without disturbing the contour of a half hardened amalgam filling or breaking off its thin corners; and when removed, it should leave this contoured filling directly and firmly in contact with the neighboring tooth, as the teeth are designed by nature. It should be easily made by the dentist or his assistant of inexpensive materials which every one can manipulate. It should not press upon or injure the gum tissues, but, by fitting itself to the tooth-walls, slip easily below the cavity margin.

If there is a matrix on the market which will fulfil all these requirements it has escaped my notice, and I have been on the lookout for such a one until recently. No material but steel fills all the requirements of a matrix, and I am at a loss to know why steel of a proper quality has not been furnished. I have found the steel used by clock-makers for the support of pendulums, when of proper gauge, to possess the desired qualities.

This steel is manufactured and sold wholesale by Jules Japy, Beaucourt, Doubs, France; but no doubt the same quality can be had at any jewellers' supply-store in America. I can only recommend exactly the gauge mentioned, six-one-hundredths millimetre, equal to twenty-three-ten-thousandths inch. A mandrel similar to that on which jewellers measure rings, but smaller, measuring fifteen millimetres in circumference at the point and forty millimetres at its larger end, divided by twenty-five lines, one-half a centimetre apart, and stamped on each line with the number corresponding to its circumference at that point, is a necessity where one desires to keep a set of matrices systematically and at hand for immediate use.

The writer has used the following method for several years, and has given several clinics with it, one of which in August, 1888, is described in the *Revue et Archives Suisses D'Odontologie,* 3ème Année, No. I.

Hood & Reynolds, of Boston, and Ash & Sons, London, make for me hard-rolled, soft gold "C" and "D" cylinders, after pattern furnished; these I use to form two-thirds of *all* gold fillings in matrices, with or without amalgam foundations. When they are used for crown fillings, they are placed upright, filling the cavity loosely, and cohesive gold is driven into the upper ends, and finished as a cohesive gold filling. The advantage is the rapid manner in which large cavities may be filled, the regular strata of gold giving great strength to the fillings. With an amalgam base in approximal cavities, the least possible retention suffices. The thin ring matrix leaves the contoured and smooth surface resting firmly on the approximal tooth, and the necessary finishing is reduced to a minimum.

The very large heavy "D" cylinders of Hood & Reynolds may also be flattened and stood edgewise on the fresh amalgam base, packed tightly, one lying flat against the other, one end resting against the matrix ring, and the other extending to the back of the cavity, and cohesive gold wedged in between these layers and built up and contoured.

The combination of gold and amalgam is probably as old as the history of amalgam, and no one claims originality in its employment. For many years we have seen occasional mention of the good results of the combination, especially the repairing of gold fillings with amalgam, and the recent agitation in its favor is owing to the fact that so few have adopted it as a regular practice. Dr. Terry, of San Remo, fills the base of deep approximal cavities with amalgam above the gum margin, and at a later sitting drills retaining points in the hardened amalgam and continues the filling as usual, with cohesive gold.

Mr. Gabriel, in the September, 1889, *Dental Record*, of London, gives in detail a method which he has used for several years. He fills the base of the deep-seated approximal molar cavity with soft copper amalgam, level with the gum margin, without matrix or rubber dam,—being assured that the retention is sufficient to hold the base quite independently of the gold to be subsequently added.

A small groove is made in the soft amalgam with a burnisher, from the buccal to the lingual side. The next day the hardened amalgam is trimmed and polished, the dam is applied, cavity dried, upper surface of filling scratched bright, and *soft* copper amalgam rubbed into the groove, and all that can be wiped away is removed. The groove now serves as a retaining point for the superstructure of gold.

The writer always prefers to use a small portion of amalgam, rubbed into the uneven surfaces of a cavity which is to be filled with soft and cohesive gold; where the artistic effect will not be marred by the exposure of the amalgam, which always becomes very dark, no matter what amalgam is used. This gives a most rigid base, and seems to anchor the gold in the most solid manner possible, and is especially useful when the retention is slight. A very slight bit of amalgam will amalgamate the base of a soft gold filling and adapt it perfectly to the floor of the cavity, and has no objectionable qualities when out of sight.

